# Antibiotic cement plate composite structure internal fixation after debridement of bone infection

**DOI:** 10.1038/s41598-021-96522-1

**Published:** 2021-08-19

**Authors:** Xiaohua Wang, Shulin Wang, Jianzhong Xu, Dong Sun, Jie Shen, Zhao Xie

**Affiliations:** Department of Orthopaedics, First Affiliated Hospital, Third Military Medical University (Army Medical University), Gaotanyan No. 30, Chongqing, 400038 The People’s Republic of China

**Keywords:** Outcomes research, Trauma

## Abstract

An internal fixation composite structure of antibiotic cement plates was created. The aim of this study was to analyse the infection control effect of this structure when applied to treat a bone infection. We retrospectively analysed patients with bone infection admitted to our hospital between January 2013 and June 2019. After debridement, an antibiotic cement plate composite structure was used to fill and stabilize the defects. The treatment effect was evaluated at six months after surgery, and the infection control rate, factors associated with the recurrence of infection, and complications were analysed. If the patients had bone defects, the defect was repaired after infection control, and the infection control rate of all of the patients was re-evaluated at 12 months after surgery. A total of 548 patients were treated with this technique, including 418 men and 130 women. The infection sites included 309 tibias, 207 femurs, 16 radii and ulnae, 13 humeri, and 3 clavicles. After at least 6 months of follow-up, 92 patients (16.79%) had an infection recurrence and needed further treatment. The recurrence rate of the tibia was higher than that of the femur (*P* = 0.025). Eighty-nine out of 92 patients who relapsed underwent a second debridement with the same method, and the infection control rate after the second debridement was 94.71%. Complications included 8 cases of epidermal necrosis around the incision, 6 cases of internal fixation failure, and 30 cases of lower limb swelling. By the follow-up time of 12 months, another 6 patients had experienced recurrence of infection, and 4 cases were controlled after debridement. Finally, among all 548 cases, 7 patients remained persistently infected, and 6 underwent amputation. The infection control rate was 97.6% at the 1-year follow-up. The clinical efficacy of this new antibiotic cement plate composite structure for internal fixation after debridement of bone infection is stable and reliable.

## Introduction

Bone infection is a devastatingly recurrent disease that often leads to limb disability or amputation, which places a heavy burden on patients, society and families^1^; it is also an important source of medical disputes. Biofilms often form on the surface of implants or sequestrum during a bone infection, especially a chronic infection, which leads to antibiotic resistance, resulting in treatment failure. Therefore, the treatment principles for bone infections are thorough debridement to remove necrotic tissue, implants and other tissues that are easily covered with biofilms, and then the application of local or systemic antibiotic treatment. Treatment of bone infection often needs to fill and stabilize the bone defects after debridement, and treatment methods such as local muscle transpositions or autologous bone grafts have solved some problems but have caused side injuries^2^ or have a high infection recurrence rate^3^. For stabilizing bone defects, external fixation has always been a classic method, but complications such as pin track infection, limited joint movement, and inconvenience limit its use^4–6^.

It has always been a wish of orthopaedic surgeons to have access to a technique that can effectively eradicate infection, be convenient to use, and reduce complications. The wide application of local antibiotics after debridement has achieved good clinical effects, but the fixation methods do not break through the limitations of traditional ideas. Some surgeons have pointed out that debridement alone is not sufficient to sterilize the operative site, and free bacteria are likely to multiply, which may cause recurrence of the infection^1^, so clinicians are extremely cautious in the use of internal fixation. Some studies have gradually explored the use of antibiotic-coated internal fixation in the treatment of bone infection^7,8^, but they mainly consist of just case reports, and no conclusive clinical evidence can be obtained.

Since 2012, our centre has specialized in the treatment and research of bone infection^9^. A composite structure of antibiotic cement plates was applied in the treatment of extremity bone infection, and the initial clinical effect was reliable^10–12^, so we named the antibiotic cement plate composite structure internal fixation for the treatment of bone infection the Chongqing technique. In this study, we applied this method to treat a large number of patients with bone infection to further analyse the infection control effect of this structure in the treatment of bone infection.

## Patients and methods

After receiving approval from the Ethics Committee of the First Affiliated Hospital, Army Medical University, and informed consent was obtained from all subjects or their parents, we retrospectively analysed the patients with bone infection admitted to our hospital between January 2013 and June 2019. All methods performed in this study were carried out in accordance with relevant guidelines and regulations. The diagnosis of bone infection was based on local bone pain and swelling, imaging procedures (X-ray, radionuclide bone scan and MRI) showing destruction or alteration of the bone, microbiological and histopathological examinations, and laboratory tests^13^. Inclusion criteria: Patients who were diagnosed with bone infection and treated with antibiotic cement plate composite structure internal fixation after debridement. The exclusion criteria: patients with no surgical treatment; patients classified as Cierny-Mader type C who were unsuitable for surgical treatment; diabetic foot infection; infection of the spine, pelvis, skull, or ribs; and patients who did not require fixation after debridement.

## Treatment methods

We evaluated the scope of debridement based on X-ray, MRI, and radionuclide bone scans before surgery. During the operation, the sinus was removed, the necrotic tissue, sequestrum and internal fixation were removed, the deep tissues were taken for bacteria isolation and pathological examination, and we collected at least 2 specimens from each lesion of the three points (core lesions, near and distal medullary). Hydrogen peroxide, diluted iodophor and normal saline were used to rinse the wound repeatedly after debridement, and the surgical staff then replaced their sterile gloves and re-sterilized the surgical site.

**Figure 1:**
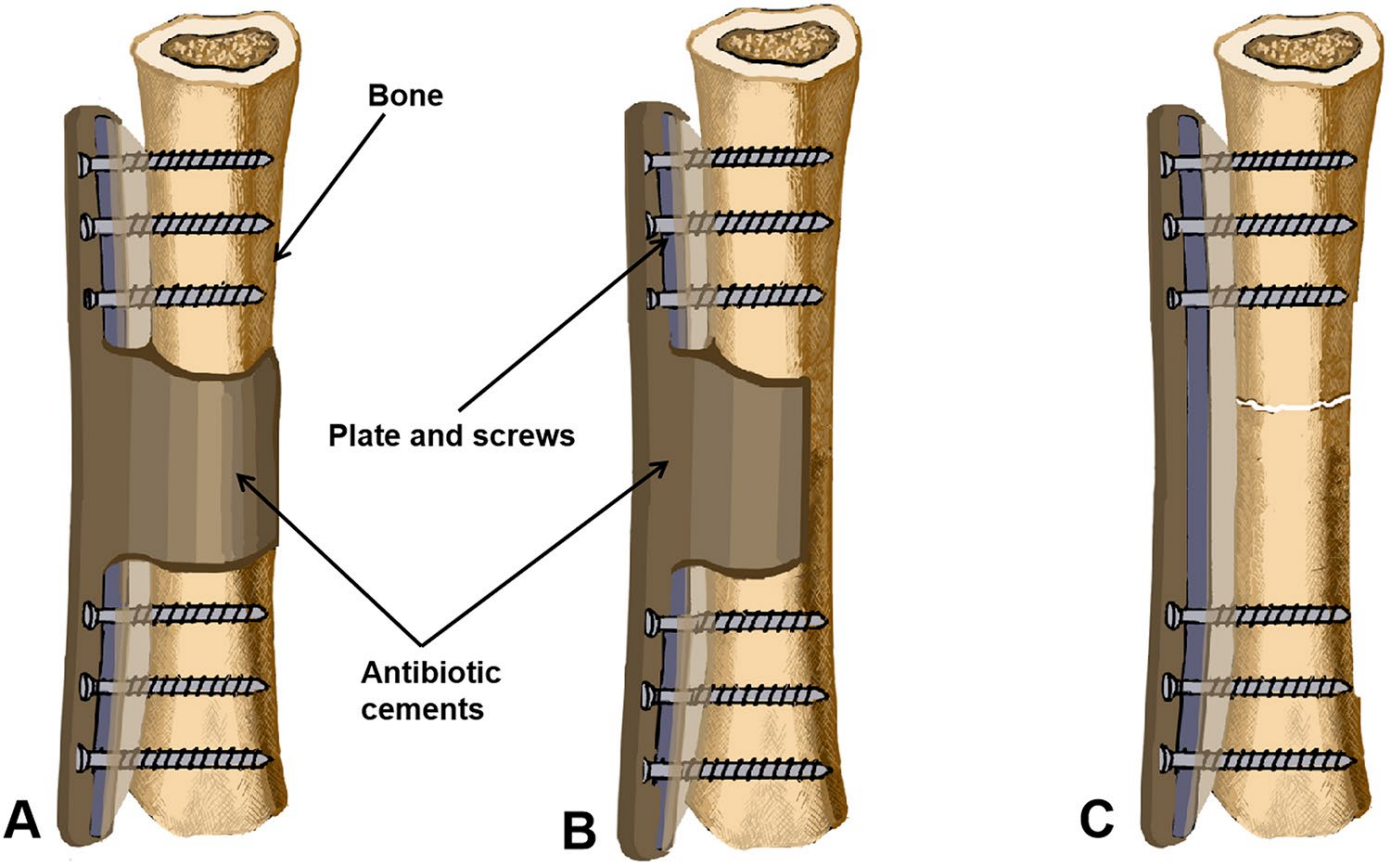
Schematic diagram of antibiotic cement plate composite structure, it is divided into three types according to the shape of bone defects. (**A**) Segmental bone defects; (**B**) partial bone defects, (**C**) no bone defect but with unhealed fracture.

After that, internal fixation was performed with a locking compression plate (Synthes, Switzerland), the bone defects were filled with antibiotic polymethyl methacrylate (PAAM) cement (5 g vancomycin was added to 40 g gentamicin cement containing 0.5 g gentamicin, Heraeus Company, Germany) and wrapped in the plate. The incision was sutured, and the chestnut was removed before the cement solidified. The composite structure was divided into three types according to the shape of the bone defect: A, segmental bone defects; B, partial bone defects; C, no bone defect but with an unhealed fracture, as shown in Fig. 1. If accompanied by intramedullary infection, an antibiotic cement stick to fill the medullary cavity was applied. If recurrence of infection occurred, debridement was performed again, and the same treatment method was adopted.

### Postoperative treatment and follow-up

**Figure 2:**
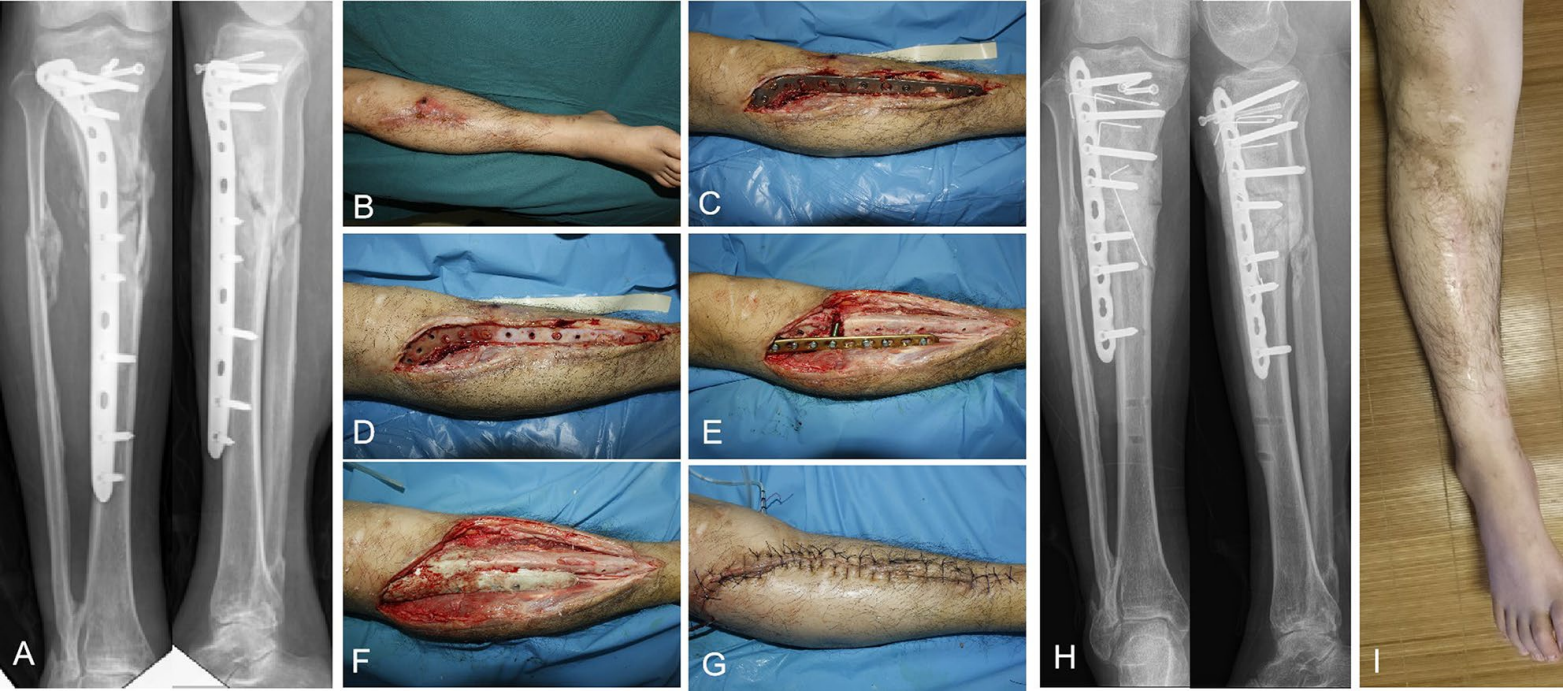
Typical case, Male, 25Y, repeatedly ulceration and pus of the right leg for 1 year, (**A**) X-rays showed bone destruction, (**B**) photo before operation, (**C**) and (**D**) infected tissue showed after removal of the internal fixation, E, bone defect was stabilized with LCP after debridement, (**F**) antibiotic cement filled the defect and wrapped the plate, (**G**) sutures the incision, (**H**) postoperative X-ray, (**I**) the incision healed at 6 months follow up.

Antibiotics were administered intravenously for 2 weeks based on bacterial isolation. Third-generation cephalosporins (ceftazidime or ceftriaxone) were routinely used before the bacterial culture results were obtained, and for those with negative results, negative pressure drainage was left in place for 10–12 days. White blood cell count (WBC), erythrocyte sedimentation rate (ESR) and C-reactive protein (CRP) were tested on the 1st, 7th, and 14th days after the operation, and whether the infection was controlled was evaluated after 6 months. The infection control criteria were as follows^14^: An apparent cure was defined as the absence of signs or symptoms of osteomyelitis for at least 6 months after therapy. Relapse was defined as infection occurring at the same site from which it was apparently eliminated, requiring specific treatment with antibiotics or surgery. A typical case is shown in Fig. 2.

According to the two-stage surgical treatment concept, additional procedures were needed to repair the defects for patients with bone defects. In the second stage, the autografts were used to fill the bone defect after infection control; if there was an insufficient source of autogenous bone, allografts were added at no more than 30% of the volume.

### Statistics

The relevant comparisons of measurement data (duration of infection, length of defect, number of debridements, etc.) were conducted using t-tests. Comparisons of the enumeration data (infection control rate, sex, smoking) were conducted using Pearson’s chi-square test. P values below 0.05 were considered significant.

## Results

A total of 548 patients were treated with this technique, including 418 men and 130 women with an average age of 40.35 ± 14.96 (4–73) years. The infection sites included 309 tibias, 207 femurs, 16 radii and ulnae, 13 humeri, and 3 clavicles. According to the Cierny-Mader classification, there were 432 type IV, 63 type III, and 47 type I cases. There were 414 traumatic and 134 haematogenous osteomyelitis cases. The average duration of infection was 71.01 ± 14.41 (1–850) months, the average number of previous operations was 2.50 ± 1.46 (0–14), 316 (57.66%) had a history of smoking, and 386 (70.44%) had skin sinuses. Among these, 389 (70.99%) had bacteria isolated, including 143 methicillin sensitive *Staphylococcus aureus* (MSSA), 45 methicillin resistant *Staphylococcus aureus* (MRSA), 31 *Pseudomonas aeruginosa*, 31 *E. coli*, 27 *E. cloacae*, 23 *Staphylococcus epidermidis*, others for 34 cases, and 55 cases with more than one bacterial species. After debridement, the average length of the defects was 7.14 ± 4.13 (0–22.5) cm. There were 312 with lacunar defects, 230 with segmental bone defects, and 6 with no defects.

After more than 6 months of follow-up, 92 patients (16.79%) relapsed after a single operation, including 64 tibia, 27 femur and 1 humerus; 89 of the 92 relapsed patients underwent a second debridement; and 60 (67.42%) had bacteria isolated. The most common bacterial species was *Staphylococcus aureus*, accounting for 28.33% (17/60). Among the relapsed patients, 19 had the same bacterial species isolated as in the first debridement, accounting for 31.7% (19/60). The infection was controlled in 63 cases, and the remaining 26 cases received a third surgical debridement.

The recurrence rate at the tibia (20.71%) was higher than that of the femur (13.04%), P = 0.025. In 386 patients with skin sinuses, the recurrence rate was 19.95% (77/386), which was significantly higher than that without sinuses, at 9.26% (15/162), *P* = 0.002. *Staphylococcus epidermidis* had the highest recurrence rate, and patients with negative bacterial isolation had the lowest recurrence rate, 39.1% (9/23) and 5% (8/159), respectively (*P* = 0.000). A total of 231 patients were classified as Cierny-Mader type A, with a recurrence rate of 16.88% (39/231), and 317 were classified as type B, with a recurrence rate of 16.72 (53/317), *P* = 0.961. There was no significant difference in the recurrence rate in terms of smoking, sex, etc. Complications included 8 cases of epidermal necrosis around the incision, 6 cases of fixation failure, and 30 cases of persistent lower extremity swelling. The results are shown in Table 1.

**Table 1:** Comparisons between the two cohorts of infection recurrent or not.

Events	Recurrent	No recurrent	*P* value
Number	92	456	–
Sex ratio (male/female)	3.60 (72/20)	3.15 (346/110)	0.624
Average age (years)	40.23 ± 15.03	40.93 ± 14.74	0.680
Patients with smoking	40.22% (37/92)	42.76% (195/456)	0.652
Cierny-Mader type (A/B)	39/53	192/264	0.960
Average duration of infection(months)	81.32 ± 16.17	68.93 ± 6.60	0.450
Patients with sinus	83.70% (77/92)	67.76% (309/456)	**0.002**
Infection site (tibia/femur)	64/27	245/180	**0.025**
Types (hematogenous/posttraumatic)	22/70	112/344	0.895
Average number of operations	2.86 ± 1.33	2.43 ± 0.85	0.51

The patients were re-evaluated for infection at 12 months follow-up. Another 6 cases had infection recurrence, and 4 cases were controlled after debridement. Finally, among all 548 cases, 7 patients remained persistently infected, and 6 underwent amputation. The infection control rate was 97.6% during the 1-year follow-up.

## Discussion

Treatment of bone infection is routinely performed by debridement and external fixation. After infection control and bone union, limb exercise is performed. Long-term external fixation is not conducive to functional exercise of the limb or to the patient's physical and mental health. At the same time, the pin track may cause infection recurrence^15^. The application of internal fixation can circumvent or reduce these complications, but internal fixation of a bone infection has always been regarded as taboo, so surgeons have been exploring the possibility of using internal fixation safely. Internal fixation with antibiotics could provide a prophylactic measure in fractures at a high risk of infection^7,16^. Thonse R used antibiotic-coated intramedullary nails for the treatment of bone infections and achieved certain clinical effects^17^. However, the production process of this technique is complicated and they are difficult to take out once the infection is controlled, which limits the popularization and application of this technique. Antibiotic bone cement to fill the medullary without fixation can achieve a high infection control rate but with a high incidence of bone non-union due to poor stability^18^. We proposed an antibiotic cement plate-composite structure to treat bone infection. The follow-up of 548 cases achieved good clinical results, and the infection control rate reached 83.21% for one debridement and 97.6% for the final result. To the best of our knowledge, this is currently the largest case series of bone infection treated with internal fixation.

Our results showed that the application of this consistent structure to treat bone infection is reliable after debridement, and the infection control rate is equivalent to a previous study^17^. However, this structure has some advantages over previous techniques. Antibiotic cement can not only fill the dead space and release antibiotics but also has a certain mechanical strength. At the same time, the structure is simple to make and can be freely shaped to fill bone defects and it is easy to remove. Functional exercise of the limb can be performed after the operation, and the patient’s ability to participate in mental and physical rehabilitation greatly shortens the healing process. The use of this structure not only avoids the disadvantages of external fixation^19^ but also disrupts the traditional treatment concept of bone infection.

The treatment of bone infection is a long process, and it is generally recommended to follow up patients for 12 months or even longer. However, studies have pointed out^14^ that bone infection assessment can be performed 6 months after surgery. In view of the fact that some of our cases required a second stage to repair their bone defects, our first follow-up was 6 months after the first surgery. Stabilization is beneficial for the treatment of bone infection^20^. Researchers have pointed out that plates may have a different susceptibility to infection^21^, so this method has a certain clinical effect in the treatment of early bone infections^22^, but for chronic infection, it is not a wise choice.

The prerequisite for internal fixation is thorough debridement^23^. At the same time, the internal fixation needs to be managed to prevent planktonic bacteria from forming biofilms on the surface of the internal fixation, which causes infection recurrence. A local antibiotic delivery system can protect not only the implant surface but also the surrounding tissues from biofilm formation^24^. No management was performed on the implants in previous studies, which resulted in high rates of recurrent infection even though the infected bone and soft tissue were removed^25^.

Thorough debridement requires comprehensive preoperative imaging and extensive bone tissue resection, and the bone resection range should be more than 5 mm^26^. At the same time, we need to pay attention to soft tissues. For the infection of surrounding soft tissues, we propose a theory of infected cyst walls, which regards the boundary between infection and normal tissue as the cyst wall; the sequestrum, pus, internal fixation, etc., are the contents of the cyst, and thus, we need to remove the contents of the cyst during debridement, and at the same time, remove the soft tissue more than 2 mm outside the cyst wall, because many bacteria, especially *Pseudomonas aeruginosa*, exhibit a particular predilection for soft tissues^9,27^. However, in clinical practice, the cyst wall is often irregular and hard to judge, so it is difficult to achieve complete debridement. Therefore, the contraindications for using this composite structure include the following: 1. Difficult to achieve complete debridement; 2. The wound is hard to close; 3. The presence of bacteria not sensitive to the antibiotics added to the bone cement.

Research has indicated that early coverage of the wound can provide nutrition, improve local immunity, and be beneficial to infection control^28^. Some people worry that the use of internal fixation will damage the local soft tissue and blood supply, which may increase the infection recurrence rate^1^. Our statistical results showed that the recurrence rate of the tibia is higher than that of the femur, and the main reason for this is that the poor skin and soft tissue condition of the lower leg delay incision healing, which leads to re-infection; our results also showed that many of these cases are not a recurrence but a re-infection^29^. Therefore, when applying this method to the tibia, it should be rich in soft tissue coverage, and the incision should not be closed forcibly to avoid adverse effects on incision healing.

Our results showed that patients with sinuses had a higher infection recurrence rate after debridement, which may be because the sinus is often not in the incision area. To ensure coverage of the wound after debridement, only limited soft tissue resection was performed. Meanwhile, patients with sinuses often have mixed infections with multiple microorganisms, making the infection more serious and more difficult to eradicate. Therefore, we thought that if the wound could be covered after debridement, the sinus should be removed as much as possible. Treatment of tibial infection should consider soft tissue coverage, fracture severity, fixation methods, and other factors that can affect its prognosis^30^. At present, the antibiotics added to bone cement are mainly gentamicin and vancomycin, and some people worry that gentamicin chain beads may cause reinfection by resistant bacteria^31^. Our results also showed a high proportion of bacteria resistant to gentamicin, and our previous study showed that patients with a bacterial infection resistant to gentamicin have a higher infection recurrence rate, especially *Pseudomonas aeruginosa* infection^32^. In the future, the types of antibiotics added to bone cement need to be further explored. At the same time, prospective studies of internal and external fixation are required for the tibia to fully verify the effectiveness and superiority of this method.

In this study, we reported the largest case series so far of bone infections treated with internal fixation and proposed a new treatment model for bone infection, but there are some shortcomings. First, the patient’s homogeneity was poor; second, it was a retrospective study. In addition, this treatment only considered infection control, and many cases required reconstruction of the defects at a later stage, and thus, the long-term treatment outcome of this method needs further investigation.
